# Supplementary material for: Leaf-venation-directed cellular alignment for macroscale cardiac constructs with tissue-like functionalities
Source: Nat Commun. 2023 Apr 12;14:2077. doi: 10.1038/s41467-023-37716-1 (PMC10097867; doi:10.1038/s41467-023-37716-1)
Supplement: Supplementary file 1 — Supplementary Information [file 41467_2023_37716_MOESM1_ESM.pdf]

Supplementary Materials for

Leaf-venation-directed cellular alignment for macroscale cardiac constructs with tissue-like functionalities

Mao Mao, Xiaoli Qu, Yabo Zhang, Bingsong Gu, Chen Li, Rongzhi Liu, Xiao Li, Hui Zhu, Jiankang He*, Dichen Li

*Corresponding author. Email: jiankanghe@mail.xjtu.edu.cn

This PDF file includes:

Supplementary Fig. 1 to 16
Supplementary Result
Supplementary Table 1

Other Supplementary Materials for this manuscript include the following:

Supplementary Movies 1 to 6

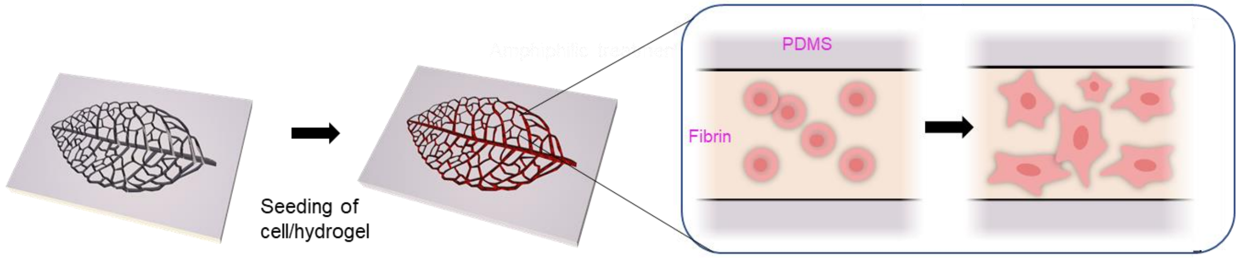

Supplementary Fig. 1.

Schematic illustration of cells/hydrogel growth within non-amphiphilic-treated PDMS.

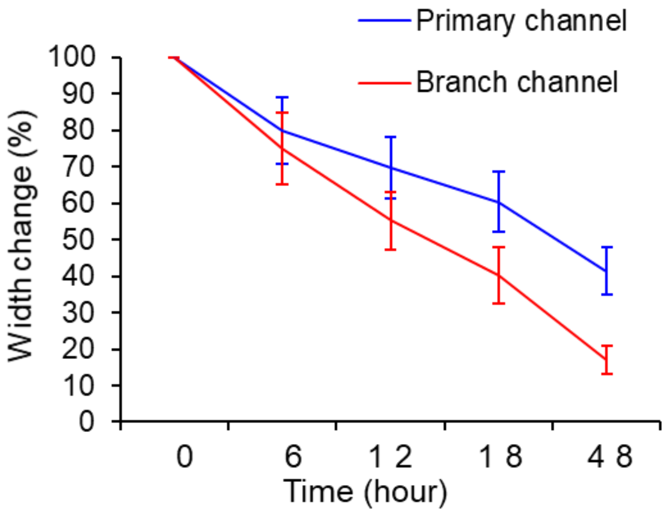

Supplementary Fig. 2.

The width change of the LVD-HUVEC tissues in the primary and branch channel. n = 4 independent samples. Data are represented as mean values ± standard deviation.

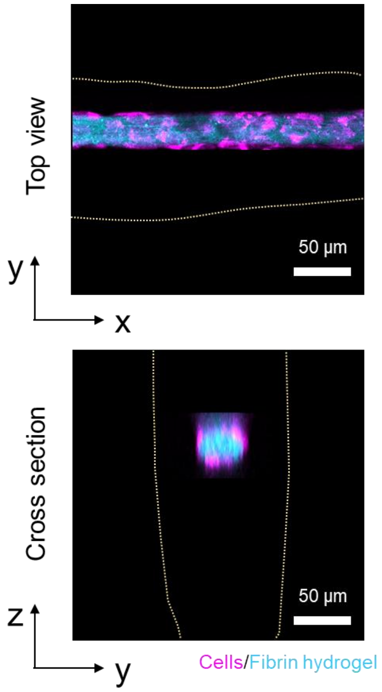

Supplementary Fig. 3.
The tubular structure of LVD-HUVEC tissues after 48 hours of culture.

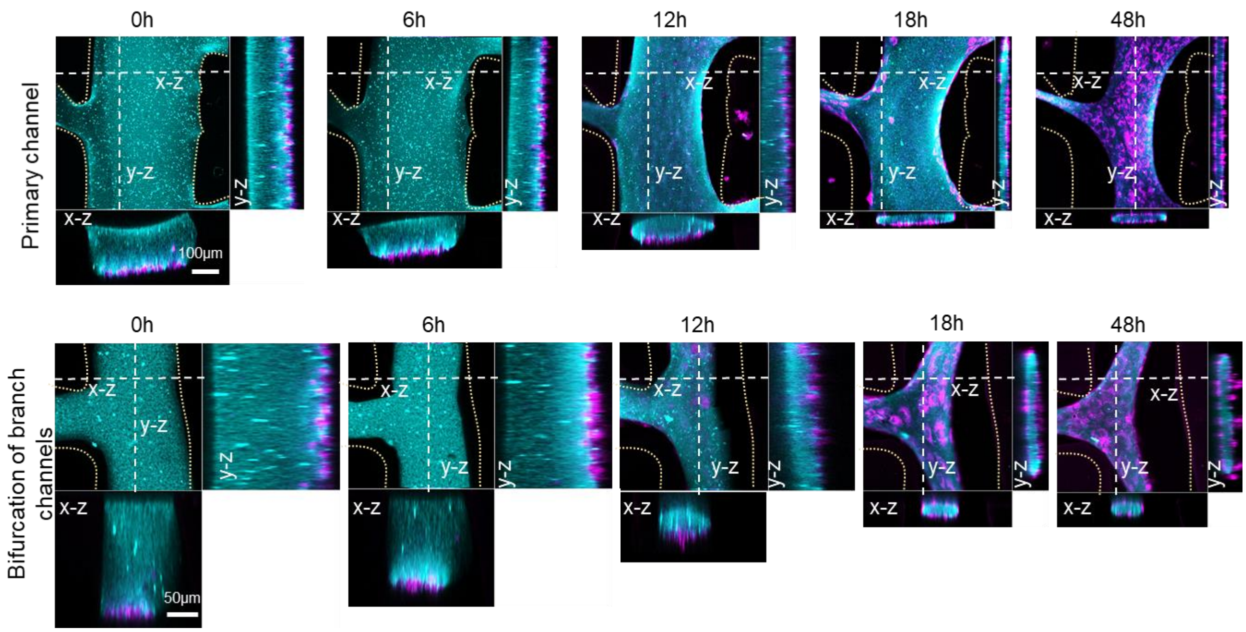

Supplementary Fig. 4.

LVD self-assembly of HUVECs (magenta)/fibrin hydrogel (cyan) into tubular structures in primary channel and bifurcation of branch channels.

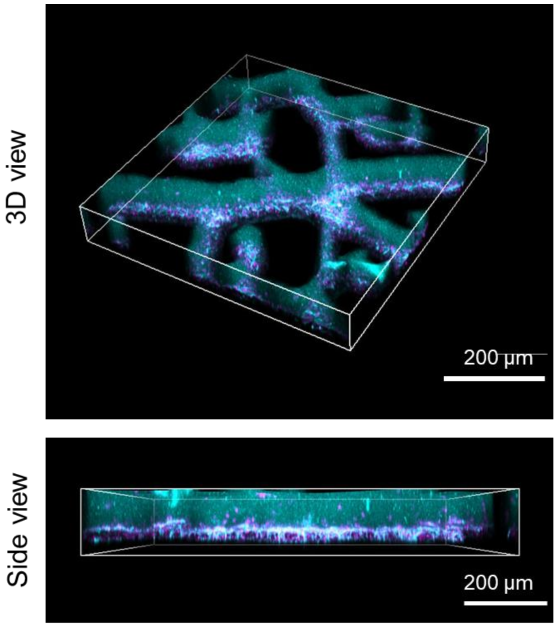

Supplementary Fig. 5.

Distribution of HUVECs (magenta)/fibrin hydrogel (cyan) in the control (non-amphiphilic-treated PDMS) after 48 hours of culture.

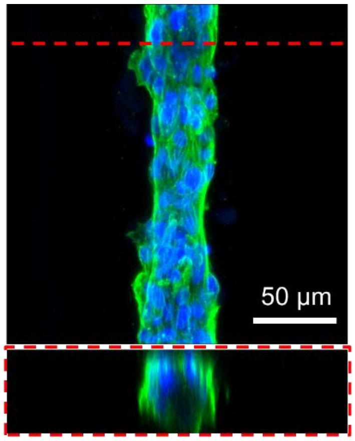

Supplementary Fig. 6.
Confocal files showing the tubular structures of LVD-cardiac tissue from hiPSC-CMs.

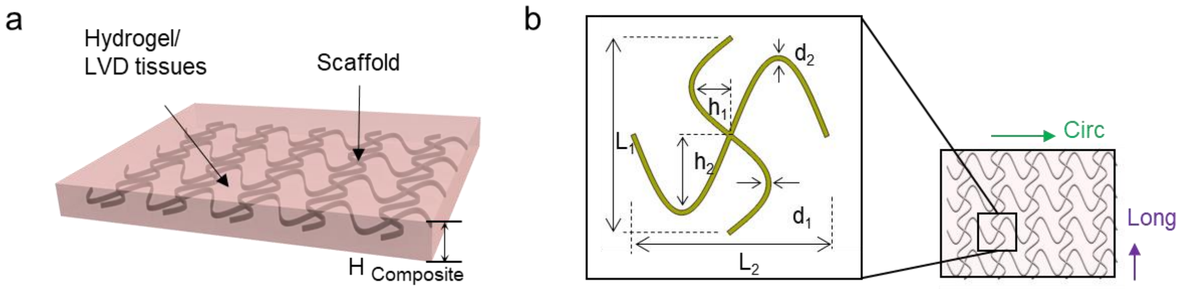

Supplementary Fig. 7.

The crucial geometrical parameters of the 3D composite tissue model for FEA calculation. For simplicity of FEA calculations, the assembled pre-vascularized cardiac constructs was taken as the 3D composite tissue model **(a)** incorporating EHD-printed PCL scaffold (elastic modulus: ~300 MPa) in soft fibrin hydrogel (elastic modulus: ~0.5 KPa), given that the enclosed LVD tissues, featuring a relatively small volume and ultra low modulus, were calculated as fibrin hydrogel. In this case, there existed several crucial geometrical parameters: w1, w2, L1, L2, H1, H2, H-scaffold, H-composite, denoting the microfiber widths along x and y directions, lengths of the unit cell along x and y directions, height of snake arc along x and y directions and thickness of the scaffold and elastomer composite respectively **(b)**.

By tuning the value of these parameters, we could independently tailor the directionally dependent mechanical property of the tissue composite along the circumferential (CIRC) and longitudinal (LONG) axes, thus providing access to tissue-like anisotropic, nonlinear mechanical properties. To predict the stress-strain curves of the rat right ventricular myocardium, a series of FEA calculations are conducted by adjusting the geometrical parameters: w1, w2, L1, L2, H1,H2, H-scaffold, H-composite, which were finally predetermined as 0.1mm, 0.1mm, 4mm, 4mm, 0.75mm, 1.6mm, 0.3mm, 0.5mm respectively through reverse design.

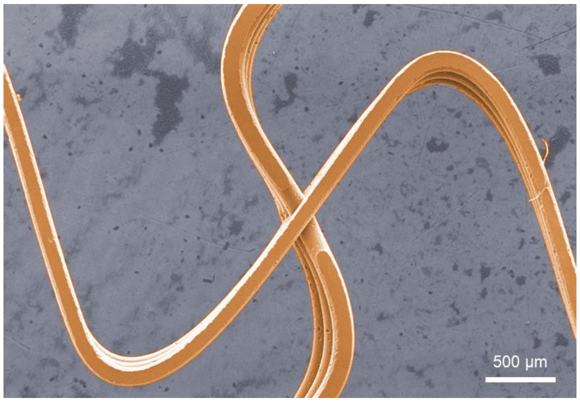

Supplementary Fig. 8.
The EHD-printed PCL scaffolds with the predesign serpentine structure.

Geometrical parameters		L1	L2	h1	h2	d1	d2	m
Designed value (mm)		4	4	0.75	1.6	0.1	0.1	0.3
Experimental value (mm)	Mean	4.015	4.013	0.787	1.638	0.114	0.123	0.326
	Standard Deviation	0.066	0.064	0.049	0.015	0.008	0.007	0.007

Supplementary Fig. 9.

The measured value of the printed scaffold's geometrical parameters, compared to the designed value.

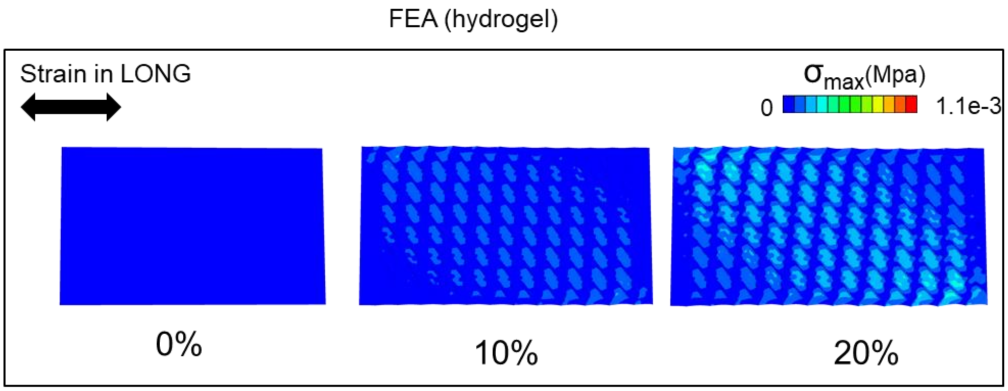

Supplementary Fig. 10.

FEA simulations of strain distribution in the scaffold of the 3D tissue composite upon uniaxial stretching (from top to bottom: 0%, 10%, and 20%) along the CIRC direction.

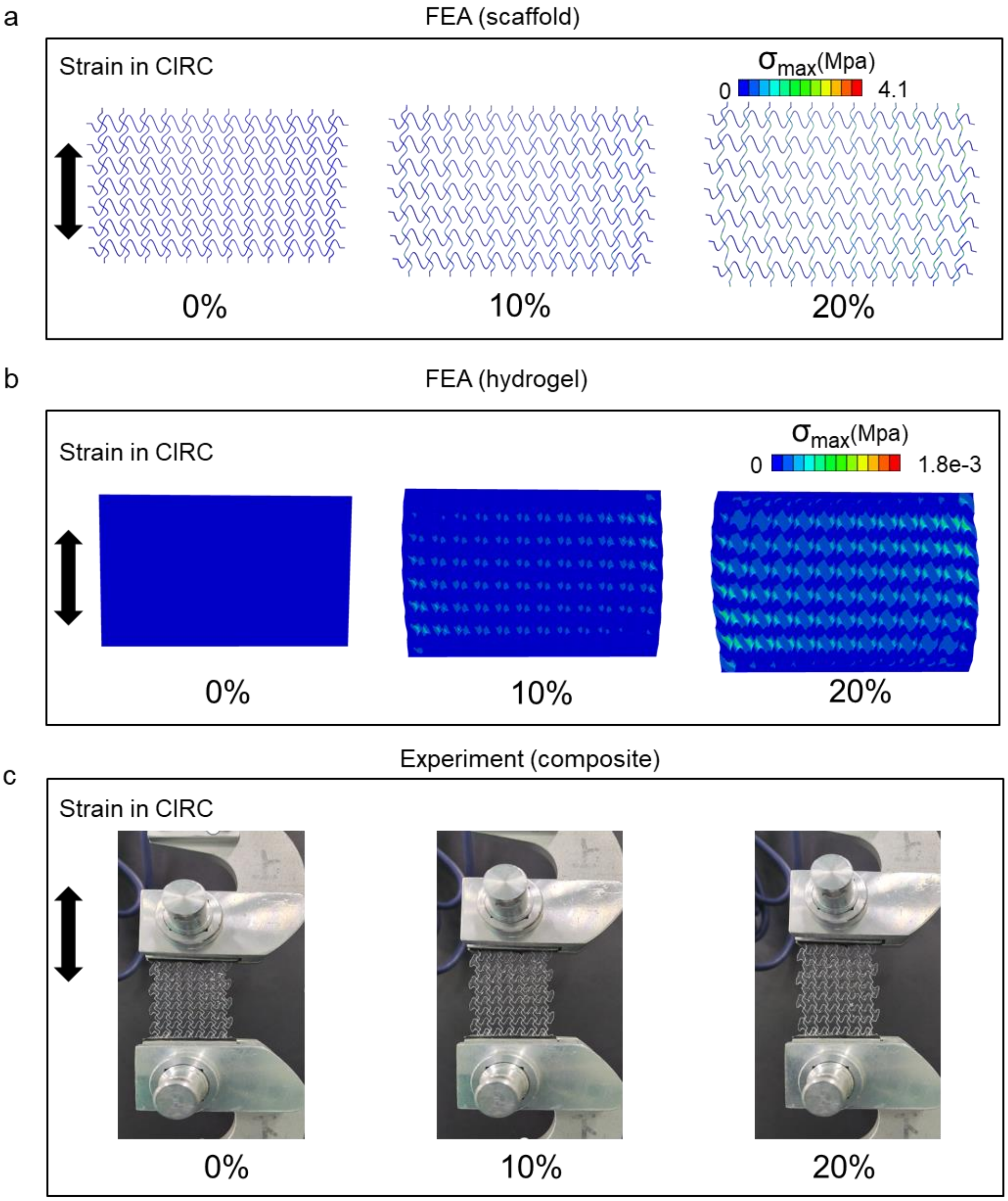

Supplementary Fig. 11.

FEA simulations and macroscopic images of the 3D tissue composites upon uniaxial stretching (from top to bottom: 0%, 10%, and 20%) along the CIRC direction. FEA simulations of strain distribution in the scaffold **(a)** and hydrogel **(b)** of the composites. **c** Macroscopic images of uniaxial stretching of the 3D tissue composites.

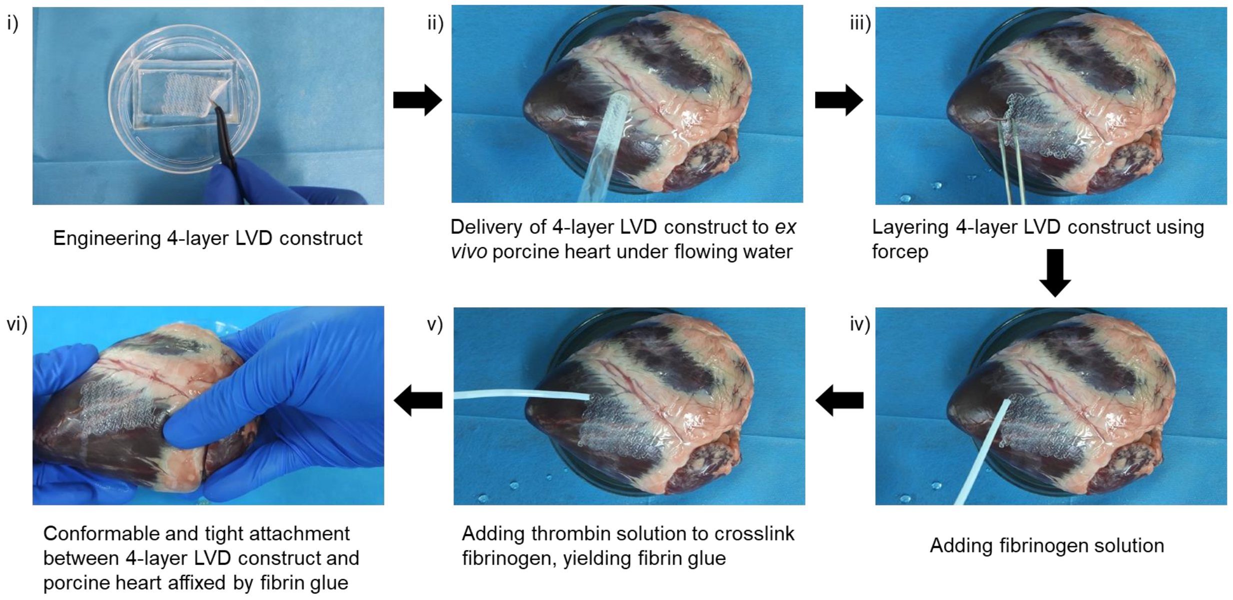

Supplementary Fig. 12.

The representative screen captures of injection and fibrin-based fixation of the LVD construct to the *ex vivo* porcine heart.

a

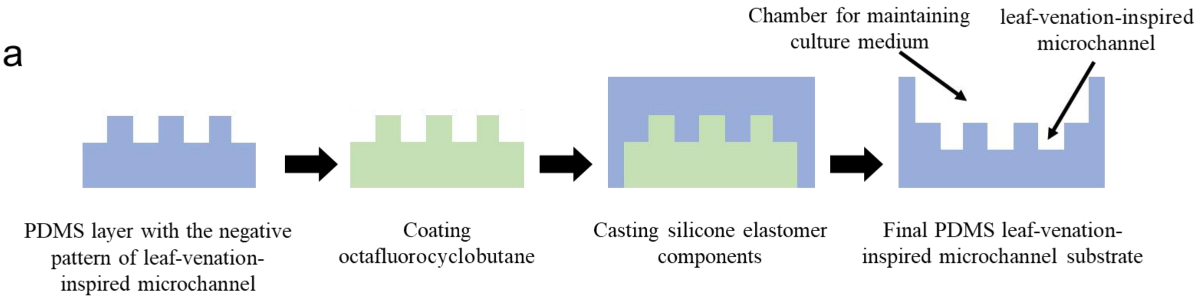

b

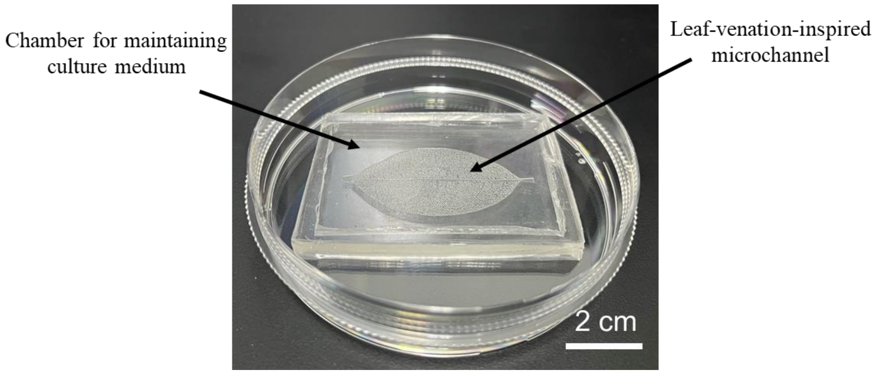

Supplementary Fig. 13.

Fabrication of PDMS leaf-venation-inspired microchannel substrate with a chamber. **a** PDMS-replication process to obtain the final PDMS leaf-venation-inspired microchannel substrate with a chamber. **b** The generated PDMS leaf-venation-inspired microchannel substrate with a chamber

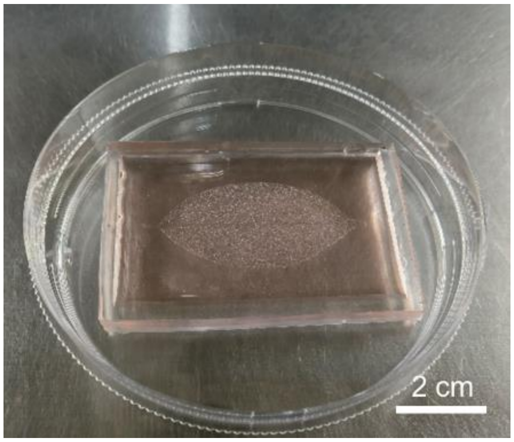

Supplementary Fig. 14.

PDMS leaf-venation-inspired microchannel substrate added with 5 ml culture medium for cell culture.

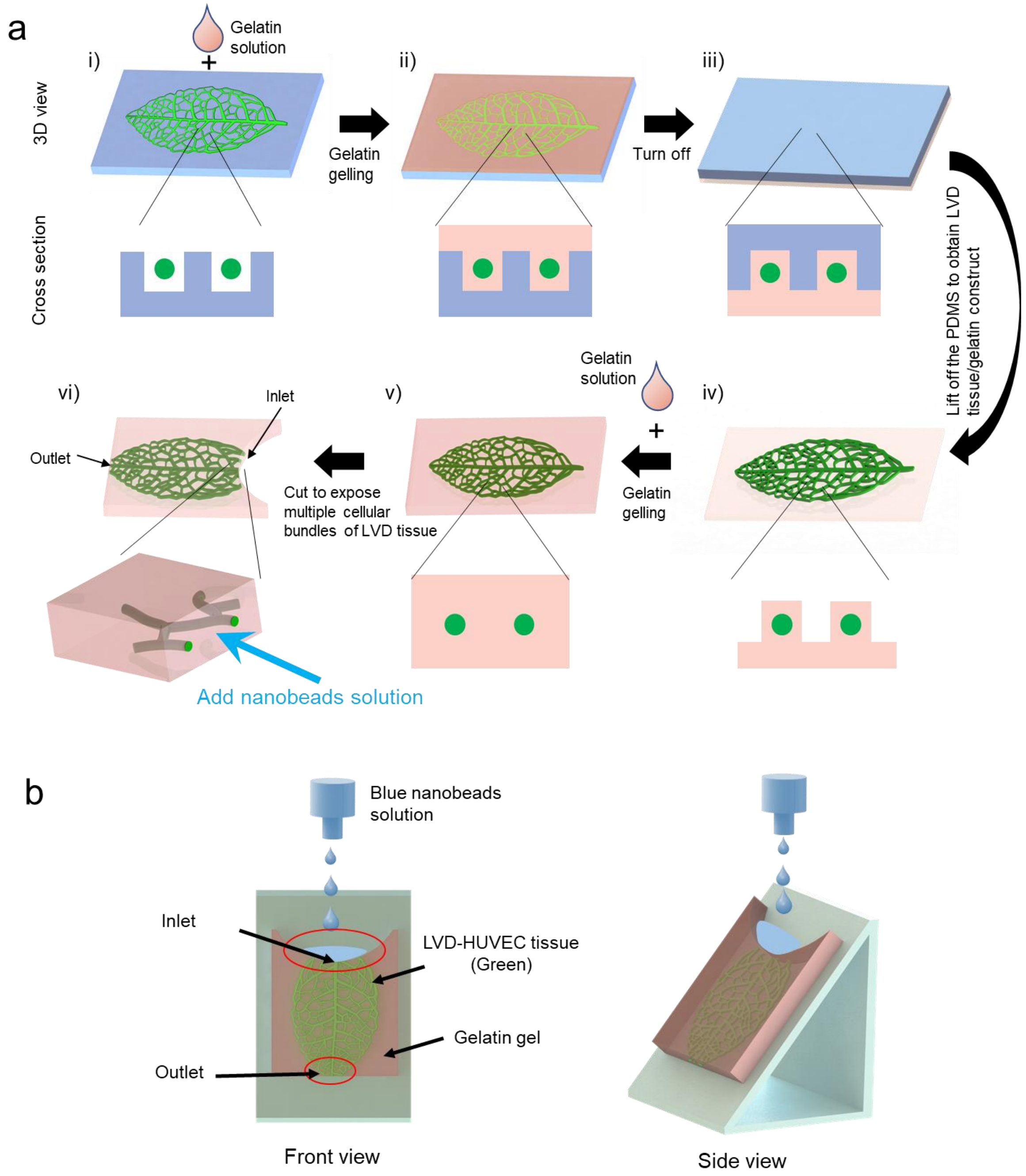

Supplementary Fig. 15.

Schematic illustration of perfusion of nanobeads solution in LVD-HUVEC tissues. **a** Generation of gelatin constructs encapsulating LVD-HUVEC networks for perfusion experiment. **b** The setup for perfusion of fluorescence nanobeads solution through LVD-HUVCE networks.

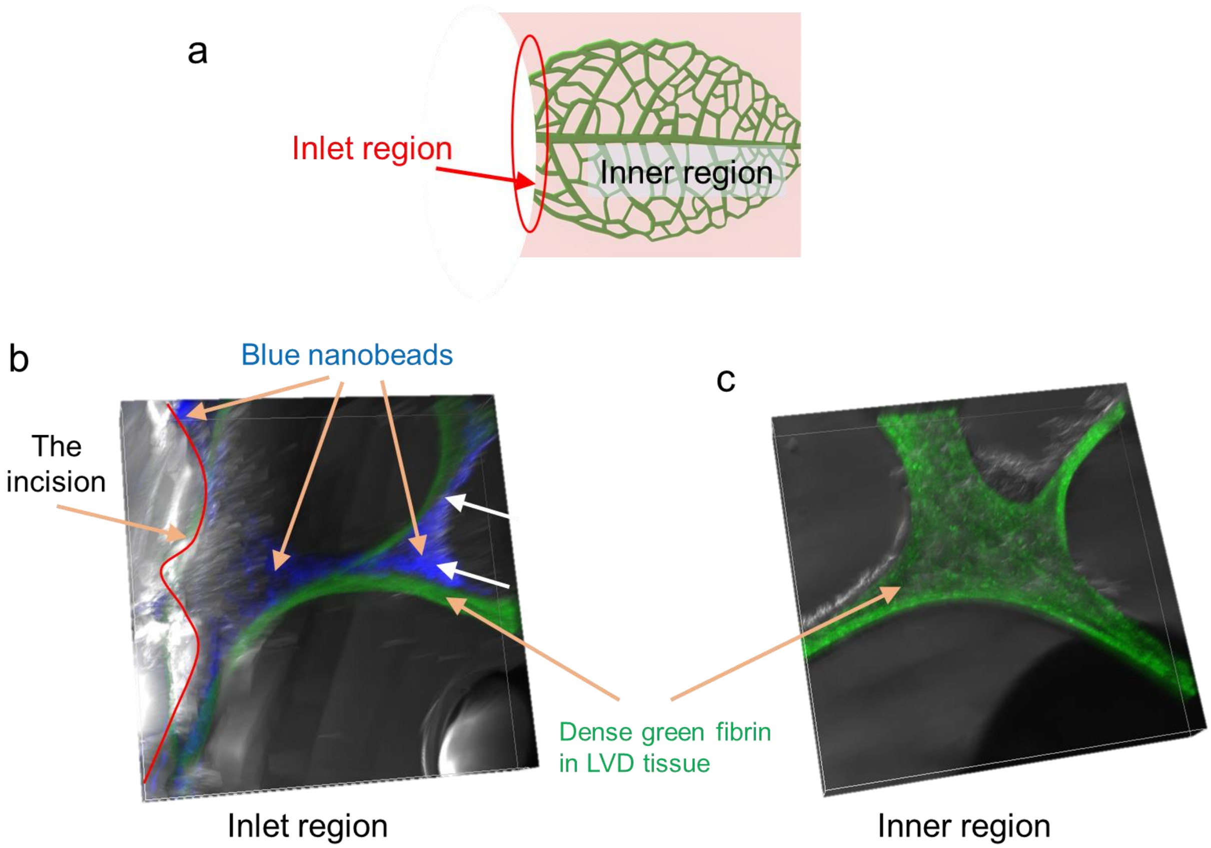

Supplementary Fig. 16.

Distribution of the perfused blue nanobeads in LVD tissue. **a** Schematic illustration of the LVD tissue/gelatin gel construct with inlet and inner regions. **b** Distribution of blue nanobeads in the inlet region. White arrows indicate some blue nanobeads in the gaps between LVD tissue (identified by dense green fibrin) and gelatin gel, which might be due to the translocation of LVD tissues during the cutting process. **c** No blue nanobeads were found in the inner region.

Supplementary Result

<u>Perfusion of nanobeads solution in LVD-HUVEC tissues</u>

Perfusion of nanobeads solution was conducted to investigate the patency of the LVD-HUVEC tissues. Specifically, when the culture medium was removed, the well-formed LVD vascular tissue in the PDMS substrate was added with 4 mL of 10 mg/mL gelatin solution (Supplementary Fig. 15ai) and placed at 4°C for 5 minutes to form a gel (Supplementary Fig. 15aii). Then, this construct was turned over (Supplementary Fig. 15aiii), and the PDMS substrate was removed, leaving the LVD vascular tissue within gelatin gel (Supplementary Fig. 15aiv). After that, another 4 mL of 5 mg/mL gelatin solution was added to them and placed at 4°C for 5 minutes, resulting in LVD vascular tissue tightly encapsulated in gelatin gel (Supplementary Fig. 15av). Finally, an arc-shaped inlet and an outlet were cut out to expose more cellular bundles for perfusing nanobeads solution (Supplementary Fig. 15avi). Then, the obtained hydrogels were placed on an inclined plane, and fluorescent nanobeads with a diameter of 80 nm (7-5-1000, Tianjin BaseLine) were pipetted to the inlet of the LVD tissue/gelatin gel construct (Supplementary Fig. 15B). Confocal images were taken at different locations inside the construct to investigate whether the nanobeads could be perfused through the LVD-HUVEC networks.

Supplementary Fig. 16 shows the representative images of nanobeads distribution in the inlet region and the inner regions. It indicates that only a few nanobeads appeared in the inlet region of the LVD tissue/gelatin gel construct (Supplementary Fig. 16b). In contrast, no nanobeads were observed in the inner regions, demonstrating the occlusion of the tubular structures of LVD-HUVEC tissue. This is in accordance with the structural assessment in Fig. 2. It can be found that the interconnected tubular structures of confluent cells in LVD-HUVEC tissue were entirely filled with the heavily-compressed fibrin fibers, which induced the occlusion of the tubular structures.

Supplementary Table 1.
List of qPCR primers.

Species	Gene	Primer
Rat	Rat-GAPDH	Forward ACAgCAACAgggTggTggAC<br>Reverse TTTgAgggTgCAgCgAACTT
	KCNJ2	Forward TgTgTTACAgACgAgTgCCC<br>Reverse CAgAgTTTgCCgTCCCTCAT
	GJA1	Forward CTCACgTCCCACggAgAAAA<br>Reverse CgCgATCCTTAACgCCTTTg
	PDK4	Forward gAgCTggTACATCCAgAgCC<br>Reverse TCgAACTTTgACCAgCgTgT
	CPT1B	Forward TCgAgTTCAgAAACgAACgC<br>Reverse gTgTgTCTCCTggTCTCAgC
	PPARGCLA	Forward ATgTgCAgCCAAgACTCTgT<br>Reverse gCTgTCTgTgTCCAggTCAT
Human	H-GAPDH-175	Forward CTCCTCCACCTTTgACgCTg<br>Reverse TCCTCTTgTgCTCTTgCTgg
	S100A1	Forward CAAggTgATgAAggAgCTAgAC<br>Reverse TCAACTgTTCTCCCAgAAgAAAT
	PLN	Forward CTCACTCgCTCAgCTATAAgAAg<br>Reverse AgAgAAgCATCACgATgATACAg
	SCN5A	Forward gAgAgAAgAAgCAAgAggAgAAA<br>Reverse CATTgCTCTgggACCATCTT
	CKM	Forward gCCgTgggCTCAgTATTT<br>Reverse ggCCTTTCTCCAACTTCTTCT
	PDK4	Forward CTgAgAATTATTgACCgCCTCT<br>Reverse gAAATTggCAAgCCgTAACC
	COX6A2	Forward gTTCCgTCCCTACCAACAC<br>Reverse gTgggCAgAgggTTCAC
