# Supplementary material for: Leaf-venation-directed cellular alignment for macroscale cardiac constructs with tissue-like functionalities
Source: Nat Commun. 2023 Apr 12;14:2077. doi: 10.1038/s41467-023-37716-1 (PMC10097867; doi:10.1038/s41467-023-37716-1)
Supplement: Supplementary file 3 — Description of additional supplementary files [file 41467_2023_37716_MOESM3_ESM.pdf]

Description of additional supplementary files

Supplementary Movie 1. Fluo-8 AM staining to demonstrate the calcium transients in the primary-channel region of LVD neonatal rat cardiac tissues after 5 days of culture.

Supplementary Movie 2. Fluo-8 AM staining to demonstrate the calcium transients in the branch-channel region of LVD neonatal rat cardiac tissues after 5 days of culture.

Supplementary Movie 3. Fluo-8 AM staining to demonstrate the calcium transients in the primary-channel region of control neonatal rat cardiac tissues after 5 days of culture.

Supplementary Movie 4. Fluo-8 AM staining to demonstrate the calcium transients in the branch-channel region of control neonatal rat cardiac tissues after 5 days of culture.

Supplementary Movie 5. Beating of LVD cardiac tissue from hiPSC-CMs after 2 days of culture.

Supplementary Movie 6. Delivery and fibrin-based fixation of 3D LVD constructs to the *ex-vivo* porcine heart.
