# Supplementary material for: Leaf-venation-directed cellular alignment for macroscale cardiac constructs with tissue-like functionalities
Source: Nat Commun. 2023 Apr 12;14:2077. doi: 10.1038/s41467-023-37716-1 (PMC10097867; doi:10.1038/s41467-023-37716-1)
Supplement: Supplementary file 10 — Reporting Summary [file 41467_2023_37716_MOESM10_ESM.pdf]

Corresponding author(s): Jiankang He

Last updated by author(s): Mar 24, 2023

Reporting Summary

Nature Portfolio wishes to improve the reproducibility of the work that we publish. This form provides structure for consistency and transparency in reporting. For further information on Nature Portfolio policies, see our Editorial Policies and the Editorial Policy Checklist.

Statistics

For all statistical analyses, confirm that the following items are present in the figure legend, table legend, main text, or Methods section.

n/a	Confirmed	
☐	☒	The exact sample size (n) for each experimental group/condition, given as a discrete number and unit of measurement
☐	☒	A statement on whether measurements were taken from distinct samples or whether the same sample was measured repeatedly
☐	☒	The statistical test(s) used AND whether they are one- or two-sided<br>*Only common tests should be described solely by name; describe more complex techniques in the Methods section.*
☒	☐	A description of all covariates tested
☒	☐	A description of any assumptions or corrections, such as tests of normality and adjustment for multiple comparisons
☐	☒	A full description of the statistical parameters including central tendency (e.g. means) or other basic estimates (e.g. regression coefficient) AND variation (e.g. standard deviation) or associated estimates of uncertainty (e.g. confidence intervals)
☐	☒	For null hypothesis testing, the test statistic (e.g. F, t, r) with confidence intervals, effect sizes, degrees of freedom and P value noted<br>*Give P values as exact values whenever suitable.*
☒	☐	For Bayesian analysis, information on the choice of priors and Markov chain Monte Carlo settings
☒	☐	For hierarchical and complex designs, identification of the appropriate level for tests and full reporting of outcomes
☒	☐	Estimates of effect sizes (e.g. Cohen's d, Pearson's r), indicating how they were calculated

Our web collection on statistics for biologists contains articles on many of the points above.

Software and code

Policy information about availability of computer code

Data collection	IS-Element AR 4.5 software was used to acquire images and videos. ABAQUS 2019 (SIMULIA, Providence RI) was used to acquire the computational simulation results.
Data analysis	Bright-field videos were analyzed for beating physiology using an open-source motion tracking software (available at https://huebschlab.wustl.edu/resources/). Fluorescent Images were analyzed using Image J (USA). All the statistical analysis was performed using Origin 2020 (OriginLab Co, Northampton, MA), MATLAB 9.4 (The Mathworks, USA) and Prism 9 (GraphPad, Inc.) software.

For manuscripts utilizing custom algorithms or software that are central to the research but not yet described in published literature, software must be made available to editors and reviewers. We strongly encourage code deposition in a community repository (e.g. GitHub). See the Nature Portfolio guidelines for submitting code & software for further information.

Data

Policy information about availability of data

All manuscripts must include a data availability statement. This statement should provide the following information, where applicable:

- Accession codes, unique identifiers, or web links for publicly available datasets
- A description of any restrictions on data availability
- For clinical datasets or third party data, please ensure that the statement adheres to our policy

All the data supporting the findings of this study are available within the main text of this article and its Supplementary Information. Source data are provided with this paper.

Human research participants

Policy information about studies involving human research participants and Sex and Gender in Research.

Reporting on sex and gender	n/a
Population characteristics	n/a
Recruitment	n/a
Ethics oversight	n/a

Note that full information on the approval of the study protocol must also be provided in the manuscript.

Field-specific reporting

Please select the one below that is the best fit for your research. If you are not sure, read the appropriate sections before making your selection.

☒ Life sciences ☐ Behavioural & social sciences ☐ Ecological, evolutionary & environmental sciences

For a reference copy of the document with all sections, see nature.com/documents/nr-reporting-summary-flat.pdf

Life sciences study design

All studies must disclose on these points even when the disclosure is negative.

Sample size	No statistical method was used to predetermine the sample size. Throughout the study, sample size was determined based on our preliminary studies and on the criteria in the field. At least 3 biological samples were included for one experiment.
Data exclusions	No data was excluded from analysis.
Replication	Experiments were repeated at least three independent experiments with similar results. All experiments were reproduced to reliably support conclusions stated in the manuscript. Experimental variation is reported in the applicable figures as standard error of the mean.
Randomization	All the tests were conducted with randomly allocated experimental groups.
Blinding	Investigators were not blinded to the sample identities during data collection since the readouts were quantitative and not prone to subjective judgment of investigators.

Reporting for specific materials, systems and methods

We require information from authors about some types of materials, experimental systems and methods used in many studies. Here, indicate whether each material, system or method listed is relevant to your study. If you are not sure if a list item applies to your research, read the appropriate section before selecting a response.

Materials & experimental systems

n/a	Involved in the study
☐	☒ Antibodies
☐	☒ Eukaryotic cell lines
☒	☐ Palaeontology and archaeology
☐	☒ Animals and other organisms
☒	☐ Clinical data
☒	☐ Dual use research of concern

Methods

n/a	Involved in the study
☒	☐ ChIP-seq
☒	☐ Flow cytometry
☒	☐ MRI-based neuroimaging

Antibodies

Antibodies used	The primary antibodies of mouse anti-sarcomeric α-actinin (ab9465), rabbit anti-CX43 (ab11370) and rabbit anti-CD31 (ab182981) were purchased from Abcam (USA). Rabbit anti-cTnt (15513-1-AP) was purchased from Proteintech (China). The secondary antibodies of Alexa Fluor 488 goat anti-mouse IgG (A11029) for α-actinin as well as Alexa Fluor 594 goat anti-rabbit IgG (A11012) for CX43 and CD31 were purchased from Invitrogen (USA). Goat anti-rabbit IgG (Alexa Flour-488) secondary antibody (ab150081) for cTnT and CD31 was purchased from Abcam (USA).
Validation	All antibodies are commercially available and have been tested by the manufacturer. Vendors and catalogue numbers are listed above, and validation information can be found on the manufacturer's website:<br>https://www.abcam.cn/sarcomeric-alpha-actinin-antibody-ea-53-ab9465.html<br>https://www.abcam.cn/cd31-antibody-epr17259-ab182981.html<br>https://www.ptgcn.com/products/TNNT2-Antibody-15513-1-AP.htm<br>https://www.abcam.cn/connexin-43--gja1-antibody-intercellular-junction-marker-ab11370.html<br>https://www.thermofisher.cn/cn/zh/antibody/product/Goat-anti-Rabbit-IgG-H-L-Cross-Adsorbed-Secondary-Antibody-Polyclonal/A-11012<br>https://www.thermofisher.cn/cn/zh/antibody/product/Goat-anti-Mouse-IgG-H-L-Highly-Cross-Adsorbed-Secondary-Antibody-Polyclonal/A-11029<br>https://www.abcam.com/goat-rabbit-igg-hl-alexa-fluor-488-preadsorbed-ab150081.html

Eukaryotic cell lines

Policy information about cell lines and Sex and Gender in Research

Cell line source(s)	HUVECs (DFSC-EC-1) were purchased from Shanghai Zhongqiaoxinzhou Biotech Co., Ltd (Shanghai, China); Neonatal rat CMs were isolated from the 2-day-old Sprague-Dawley rats according to the guide for the care and use of laboratory animals at Xi'an Jiaotong University. hiPSC-CMs (HELP4111, NovoCellTM) were purchased from Help Therapeutics (Nanjing, China)
Authentication	Neonatal rat CMs were analyzed by immunostaining for CM makers α-actinin and CX43. HUVECs and hiPSC-CMs was used as received from the supplier.
Mycoplasma contamination	All cell lines tested negative for mycoplasma contamination.
Commonly misidentified lines (See ICLAC register)	There were no misidentified cells in our study.

Animals and other research organisms

Policy information about studies involving animals; ARRIVE guidelines recommended for reporting animal research, and Sex and Gender in Research

Laboratory animals	2-day-old Sprague–Dawley rats were purchased from Laboratory Animal Center of Xi'an Jiaotong University for isolation of neonatal rat cardiomyocytes. ~30 rats were used for the cell isolation.
Wild animals	The study did not involve wild animals.
Reporting on sex	This study did not involve sex-based design and analysis. In this study, the neonatal rat cardiomyocytes were isolated from the 2-day-old Sprague-Dawley rats that are quite immature, and gender differences would not affect the produced cardiomyocytes.
Field-collected samples	This study did not involve samples collected from the field.
Ethics oversight	All animal procedures were performed according to the guide for the care and use of laboratory animals at Xi’an Jiaotong University and were approved by Animal Ethics Committee at Xi'an Jiaotong University (Approval numbers: 2021-1242).

Note that full information on the approval of the study protocol must also be provided in the manuscript.
